# Higgs characterisation via vector-boson fusion and associated production: NLO and parton-shower effects

**DOI:** 10.1140/epjc/s10052-013-2710-5

**Published:** 2014-01-25

**Authors:** Fabio Maltoni, Kentarou Mawatari, Marco Zaro

**Affiliations:** 1 Centre for Cosmology, Particle Physics and Phenomenology (CP3), Université Catholique de Louvain, 1348 Louvain-la-Neuve, Belgium; 2 Theoretische Natuurkunde and IIHE/ELEM, Vrije Universiteit Brussel, and International Solvay Institutes, Pleinlaan 2, 1050 Brussels, Belgium; 3 LPTHE, CNRS UMR 7589, UPMC Univ. Paris 6, 75252 Paris, France

## Abstract

Vector-boson fusion and associated production at the LHC can provide key information on the strength and structure of the Higgs couplings to the Standard Model particles. Using an effective field theory approach, we study the effects of next-to-leading order (NLO) QCD corrections matched to a parton shower on selected observables for various spin-0 hypotheses. We find that inclusion of NLO corrections is needed to reduce the theoretical uncertainties on the total rates as well as to reliably predict the shapes of the distributions. Our results are obtained in a fully automatic way via FeynRules and MadGraph5_aMC@NLO.

## Introduction

After the discovery of a new boson at the LHC [1, 2], studies of its properties have become the first priority of the high-energy physics community. A coordinated theoretical and experimental effort is made [3–5] that aims at maximising the information from the ongoing and forthcoming measurements. On the experimental side, new analyses, strategies and more precise measurements are being performed that cover the wider range of relevant production and decay channels in the Standard Model (SM) and beyond, and the recent measurements of the coupling strength [6, 7] and the spin-parity properties [8, 9] give strong indications that the new particle is indeed the scalar boson predicted by the SM. On the theoretical side, predictions for signal and background are being obtained at higher orders in perturbative expansion in QCD and electroweak (EW) theory, so that a better accuracy in the extraction of the SM parameters can be achieved. In addition, new variables and observables are being proposed that may be sensitive to new physics effects. At the same time, considerable attention is being devoted to the definition of a theoretical methodology and framework to collect and interpret the constraints coming from the experimental side.

The proposal of employing an effective field theory (EFT) that features only SM particles and symmetries at the EW scale has turned out to be particularly appealing. Such a minimal assumption, certainly well justified by the present data, provides not only a drastic reduction of all possible interactions that Lorentz symmetry alone would allow, but also a well-defined and powerful framework where constraints coming from Higgs measurements can be globally analysed together with those coming from precision EW measurements and flavour physics (see for example Refs. [10–34], and more in general Refs. [35, 36]).

In this context, the *Higgs Characterisation* (HC) framework has recently been presented [37], which follows the general strategy outlined in Ref. [38]. A simple EFT lagrangian featuring bosons with various spin-parity assignments has been implemented in FeynRules [39, 40] and passed to the MadGraph5_aMC@NLO [41–43] framework by means of the UFO model file [44, 45]. Such an implementation is simple but general enough to describe any new physics effects coming from higher scales in a fully model-independent way. It has the advantage of being systematically and seamlessly improvable through the inclusion of more operators in the lagrangian on one side and of higher-order corrections, notably those coming from QCD, on the other. The latter, considered in the form of multi-parton tree-level computations (ME+PS) and of next-to-leading order (NLO) calculations matched to parton showers (NLO+PS), are a very important ingredient for performing sensible phenomenological studies.

In Ref. [37] we have provided a study of higher-order QCD effects for inclusive $pp\rightarrow X(J^P)$ production, with $J^P=0^+$, $0^-$, $1^+$, $1^-,$ and $2^+$, and correlated decay of resonances into a pair of gauge bosons, where gluon fusion ($q{\bar{q}}$ annihilation) is dominant for spin-0 and spin-2 (spin-1) at the LO. In this work, we present the results for the next most important production channels at the LHC, *i.e.*, weak vector-boson fusion (VBF) and associated production (VH), focusing on the most likely spin-0 hypothesis. As already noted in Ref. [37], these processes share the property that NLO QCD corrections factorise exactly with respect to the new physics interactions in Higgs couplings and therefore can be automatically performed within the current MadGraph5_aMC@NLO framework. Given that the Higgs characterisation can also be done automatically in the $t{\bar{t}}H$ production channel [46], all the main Higgs production channels are covered.

We stress that the spin-parity studies in VBF and VH production nicely complement those in $H\rightarrow ZZ/WW$ decays [47, 48]. One of the advantages in the VBF and VH channels is that spin-parity observables, *e.g.*, the azimuthal difference between the two tagging jets $\varDelta \phi _{jj}$ in VBF, do not require a reconstruction of the Higgs resonance, although the separation between the $Z$ and $W$ contributions is very difficult. In this study, we focus on the effects of the QCD corrections in Higgs VBF and VH production without considering the decay.

The paper is organised as follows. In the following section we recall the relevant effective lagrangian of Ref. [37], and we define the sample scenarios used to illustrate the phenomenological implications. In Sect. 3 we present the VBF results in the form of distributions of key observables in the inclusive setup as well as with dedicated VBF cuts, while in Sect. 4 we illustrate the $W^{\pm }H$ and $ZH$ production. We briefly summarise our findings in the concluding section.

## Theoretical setup

In this section, we summarise the full setup, from the lagrangian, to the choice of benchmark scenarios, to event generation at NLO accuracy.

### Effective lagrangian and benchmark scenarios

We construct an effective lagrangian below the electroweak symmetry breaking (EWSB) scale in terms of mass eigenstates. Our assumptions are simply that the resonance structure observed in data corresponds to one bosonic state ($X(J^P)$ with $J=0$, $1$ or $2$ and a mass of about $125$ GeV), and that no other new state below the cutoff $\varLambda$ coupled to such a resonance exists. We also follow the principle that any new physics is dominantly described by the lowest-dimensional operators. This means, for the spin-0 case, that we include all effects coming from the complete set of dimension-six operators with respect to the SM gauge symmetry.

The effective lagrangian relevant for this work, *i.e.*, for the interactions between a spin-0 state and vector bosons, is (Eq. (2.4) in Ref. [37]):

1 $$\begin{aligned} {\mathcal {L}}_0^{V}&=\left\{ c_{\alpha }\kappa _\mathrm{SM}\left[ \frac{1}{2}g_{\scriptscriptstyle HZZ}\, Z_\mu Z^\mu +g_{\scriptscriptstyle HWW}\, W^+_\mu W^{-\mu }\right] \right. \nonumber \\&\quad -\frac{1}{4}\left[ c_{\alpha }\kappa _{\scriptscriptstyle H\gamma \gamma } g_{\scriptscriptstyle H\gamma \gamma } \, A_{\mu \nu }A^{\mu \nu } +s_{\alpha }\kappa _{\scriptscriptstyle A\gamma \gamma }g_{ \scriptscriptstyle A\gamma \gamma }\, A_{\mu \nu }{\widetilde{A}}^{\mu \nu } \right] \nonumber \\&\quad -\frac{1}{2}\left[ c_{\alpha }\kappa _{\scriptscriptstyle HZ\gamma }g_{\scriptscriptstyle HZ\gamma } \, Z_{\mu \nu }A^{\mu \nu } +s_{\alpha }\kappa _{\scriptscriptstyle AZ\gamma }g_{\scriptscriptstyle AZ\gamma }\,Z_{\mu \nu }{\widetilde{A}}^{\mu \nu } \right] \nonumber \\&\quad -\frac{1}{4}\left[ c_{\alpha }\kappa _{\scriptscriptstyle Hgg}g_{\scriptscriptstyle Hgg} \, G_{\mu \nu }^aG^{a,\mu \nu } +s_{\alpha }\kappa _{\scriptscriptstyle Agg}g_{\scriptscriptstyle Agg}\,G_{\mu \nu }^a{\widetilde{G}}^{a,\mu \nu } \right] \nonumber \\&\quad -\frac{1}{4}\frac{1}{\varLambda }\left[ c_{\alpha }\kappa _{\scriptscriptstyle HZZ} \, Z_{\mu \nu }Z^{\mu \nu } +s_{\alpha }\kappa _{\scriptscriptstyle AZZ}\,Z_{\mu \nu }{\widetilde{Z}}^{\mu \nu } \right] \nonumber \\&\quad -\frac{1}{2}\frac{1}{\varLambda }\left[ c_{\alpha }\kappa _{\scriptscriptstyle HWW} \, W^+_{\mu \nu }W^{-\mu \nu } +s_{\alpha }\kappa _{\scriptscriptstyle AWW}\,W^+_{\mu \nu }{\widetilde{W}}^{-\mu \nu }\right] \nonumber \\&\quad -\frac{1}{\varLambda }c_{\alpha } \left[ \kappa _{\scriptscriptstyle H\partial \gamma } \, Z_{\nu }\partial _{\mu }A^{\mu \nu } \kappa _{\scriptscriptstyle H\partial Z} \, Z_{\nu }\partial _{\mu }Z^{\mu \nu }\right. \nonumber \\&\quad \left. \left. + \, \left( \kappa _{\scriptscriptstyle H\partial W} W_{\nu }^+\partial _{\mu }W^{-\mu \nu }+h.c.\right) \right] \right\} X_0\,, \end{aligned}$$

where the (reduced) field strength tensors are defined as

2 $$\begin{aligned} V_{\mu \nu }&=\partial _{\mu }V_{\nu }-\partial _{\nu }V_{\mu }\quad (V=A,Z,W^{\pm })\,, \end{aligned}$$

3 $$\begin{aligned} G_{\mu \nu }^a&=\partial _{\mu }^{}G_{\nu }^a-\partial _{\nu }^{}G_{\mu }^a +g_sf^{abc}G_{\mu }^bG_{\nu }^c\,, \end{aligned}$$

and the dual tensor is

4 $$\begin{aligned} {\widetilde{V}}_{\mu \nu } =\frac{1}{2}\epsilon _{\mu \nu \rho \sigma }V^{\rho \sigma }. \end{aligned}$$

Our parametrisation: (i) allows one to recover the SM case easily by the dimensionless coupling parameters $\kappa _i$ and the dimensionful couplings $g_{\scriptscriptstyle Xyy'}$ shown in Tables 1 and 2; (ii) includes $0^-$ state couplings typical of SUSY or of generic two-Higgs-doublet models (2HDM); (iii) describes CP-mixing between $0^+$ and $0^-$ states, parametrised by an angle $\alpha$, in practice $-1<c_{\alpha }\,(\equiv \cos \alpha )<1$.

**Table 1:** HC model parameters

Parameter	Description
$\varLambda$ (GeV)	Cutoff scale
$c_{\alpha }\,(\equiv \cos \alpha$)	Mixing between $0^+$ and $0^-$
$\kappa _i$	Dimensionless coupling parameter

**Table 2:** Values in units of $v$ taken by the couplings $g_{Xyy'}$ for the EW gauge bosons. $C=\sqrt{\frac{\alpha _{\scriptscriptstyle \mathrm EM}G_F m_Z^2}{8\sqrt{2}\pi }}$

$$g_{Xyy'}\times v\ \ $$	$ZZ/WW$	$\gamma \gamma$	$Z\gamma$
$X=H$	$2m_{Z/W}^2$	$47\alpha _{\scriptscriptstyle \mathrm EM}/18\pi$	$C(94c^2_W-13)/9\pi$
$X=A$	0	$4\alpha _{\scriptscriptstyle \mathrm EM}/3\pi$	$2C(8c^2_W-5)/3\pi$

The corresponding implementation of the dimension-six lagrangian above the EWSB scale, where $SU(2)_L \times U(1)_Y$ is an exact symmetry, has recently appeared [49], which has overlapping as well as complementary features with respect to our HC lagrangian. We note that the lagrangian of Eq. (1) features 14 free parameters, of which one possibly complex ($\kappa _{\scriptscriptstyle H\partial W}$). On the other hand, as explicitly shown in Table 1 of Ref. [49] these correspond to 11 free parameters in the parametrisation above the EWSB due to the custodial symmetry. We stress that the results at NLO in QCD accuracy shown here can be obtained for that lagrangian in exactly the same way.

In Table 3 we list the representative scenarios that we later use for illustration. The first corresponds to the SM. The second scenario, $0^+$(HD), includes only the CP-even higher-dimensional operators corresponding to $\kappa _{\scriptscriptstyle HZZ,HWW}$ in a custodial invariant way for VBF. The third scenario, $0^+$ (HDder), includes the so-called derivative operators which, via the equations of motions, can be linked to contact operators of the type $HVff'$. The fourth scenario, $0^+$(SM+HD), features the interference, which scales as $1/\varLambda$ in the physical observables, between the SM and the HD operators. The fifth scenario, $0^-$(HD), is the analogous of the second one, but for a pseudoscalar. Finally, the sixth scenario, $0^\pm$(HD), is representative of a CP-mixed case, where the scalar is a scalar/pseudoscalar state in equal proportion.

**Table 3:** Benchmark scenarios

Scenario	HC parameter choice
$0^+$(SM)	$\kappa _{\scriptscriptstyle \mathrm SM}=1\ (c_{\alpha }=1)$
$0^+$(HD)	$\kappa _{\scriptscriptstyle HZZ,HWW}=1\ (c_{\alpha }=1)$
$0^+$(HDder)	$\kappa _{\scriptscriptstyle H\partial Z,H\partial W}=1\ (c_{\alpha }=1)$
$0^+$(SM$+$HD)	$\kappa _{\scriptscriptstyle SM,HZZ,HWW}=1\ (c_{\alpha }=1,\, \varLambda =v)$
$0^-$(HD)	$\kappa _{\scriptscriptstyle AZZ,AWW}=1\ (c_{\alpha }=0)$
$0^{\pm }$(HD)	$\kappa _{\scriptscriptstyle HZZ,AZZ,HWW,AWW}=1\ (c_{\alpha }=1/\sqrt{2})$

### NLO corrections including parton-shower effects

The MadGraph5_aMC@NLO framework is designed to automatically perform the computation of tree-level and NLO cross sections, possibly including their matching to parton showers and the merging of samples with different parton multiplicities. Currently, the full automation is available in a unique and self-contained framework based on MadGraph5 [41] for SM processes with NLO QCD corrections. User intervention is limited to the input of physical quantities, and after event generation, to the choice of observables to be analysed. In Ref. [37] the results for gluon fusion have been presented and compared to predictions coming from ME+PS (MLM-$k_T$ merging [50–52]) and NLO +PS. The distributions were found to be compatible between the two predictions. In this work we limit ourselves to NLO+PS results as typical observables are inclusive in terms of extra radiation and such calculations do also provide a reliable normalisation.

aMC@NLO implements matching of any NLO QCD computation with parton showers following the MC@NLO approach [53]. Two independent and modular parts are devoted to the computation of specific contributions to an NLO-matched computation: MadFKS [42] takes care of the Born, the real-emission amplitudes, and it also performs the subtraction of the infrared singularities and the generation of the Monte Carlo subtraction terms, according to the FKS prescription [54, 55]. MadLoop [43] computes the one-loop amplitudes, using the CutTools [56] implementation of the OPP integrand-reduction method [57]. The OpenLoops method [58] is also used for better performance. Once the process of interest is specified by the user, the generation of the code is fully automated. Basic information, however, must be available as regards the model and the interactions of its particles with QCD partons. For MadFKS this amounts to the ordinary Feynman rules. For MadLoop, on the other hand, the Feynman rules, UV counterterms, and special tree-level rules, so-called $R_2$, necessary to (and defined by) the OPP method, should be provided. While Feynman rules are automatically computed from a given lagrangian (via FeynRules [39, 40]), this is not yet possible for UV counterterms and $R_2$ rules. At this moment this limitation hampers the automatic computation of NLO QCD corrections for arbitrary processes in generic BSM models, including the HC model. The processes considered in this paper, VBF and VH, are, however, a notable exception as QCD corrections can be computed automatically and in full generality. This is because the corresponding one-loop amplitudes only include SM particles and do not need any UV counterterms and $R_2$ information from the HC lagrangian. In the case of VBF, this assumes that only vertex loop corrections can be computed, *i.e.*, the pentagon diagrams are discarded, as the contributions only affect interferences between the diagrams, which are negligible already at LO.

### Simulation parameters

In our simulations we generate events at the LHC with a centre-of-mass energy $\sqrt{s}=8$ TeV and set the resonance mass to $m_{X_0}=125$ GeV. Parton distribution functions (PDFs) are evaluated by using the MSTW2008 (LO/NLO) parametrisation [59], and jets are reconstructed via the anti-$k_T$ ($\varDelta R=0.4$) algorithm [60] as implemented in FastJet [61]. Central values for the renormalisation and factorisation scales $\mu _{R,F}$ are set to $\mu _0=m_W$ and $m_\mathrm{VH}$ for VBF and VH production, respectively, where $m_\mathrm{VH}$ is the invariant mass of the VH system. We note here that scale (and PDF) uncertainties can be evaluated automatically in the code via a reweighting technique [62], the user only deciding the range of the variation. In addition, such information is available on an event-by-event basis and therefore uncertainty bands can be plotted for any observable of interest. In this work, however, to simplify the presentation that focuses on the differences between the various scenarios, we give this information only for total cross sections and refrain from showing them in the differential distributions. For parton shower and hadronisation we employ HERWIG6 [63] in this paper, while HERWIG++ [64], (virtuality ordered) Pythia6 [65] and Pythia8 [66] are available for use in aMC@NLO. The comparison among the above different shower schemes was done for the SM Higgs boson in VBF in Ref. [67].

## Vector-boson fusion

Predictions for Higgs production via VBF in the SM are known up to NNLO accuracy for the total cross section [68–70], at the NLO QCD [71–76] + EW [77, 78] level in a differential way and at NLO in QCD plus parton shower both in the POWHEG BOX [79] and in aMC@NLO [67]. NLO QCD predictions that include anomalous couplings between the Higgs and a pair of vector bosons are available in VBFNLO [80, 81]. Our implementation provides the first predictions for EFT interactions including NLO corrections in QCD interfaced with a parton shower. Many phenomenological studies on Higgs spin, parity and couplings are available in the literature [47, 48, 82–88], which could now be upgraded to NLO+PS accuracy.

In our framework the code and events for VBF can be automatically generated by issuing the following commands (note the $$ sign to forbid diagrams with $$W^{\pm }$$ or $$Z$$ bosons in the $$s$$-channel which are included in VH production): 
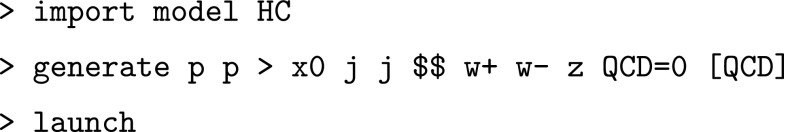
 As a result all processes featuring a $$VV^\prime \rightarrow X_0$$ vertex, with $$V=W,Z,\gamma $$, are generated, therefore including $$\gamma \gamma \rightarrow X_0$$ and $$Z\gamma \rightarrow X_0$$. We do not investigate their effects in our illustrative studies below (*i.e.*, we set the corresponding $$\kappa _i$$ to zero in the simulation), as we focus on SM-like VBF observables. As mentioned above, since our interest is geared towards QCD effects on production distributions, we do not include Higgs decays in our studies either. We stress, however, that decays (as predicted in the HC model) can be efficiently included at the partonic event level (before passing the event to a shower program) via MadSpin [89].

In Table 4, we first collect results for total cross sections at LO and NLO accuracy together with scale uncertainties and corresponding $$K$$-factors for the six scenarios defined in Table 3. We do not impose any cuts here, and hence the cross sections are identical with and without parton shower. The cross sections for the HD hypotheses are calculated with the corresponding $$\kappa _i$$ set to 1 and the cutoff scale $$\varLambda =1$$ TeV except for the $$0^+$$(SM+HD) scenario, where we set $$\varLambda =v=246$$ GeV. We do this to allow for visible effects of the interference between the SM and HD terms. Equivalently, we could have kept $$\varLambda =1$$ TeV and chosen a larger value for $$\kappa _i$$, as only the ratio $$\kappa _i/\varLambda $$ is physical. The figures in parentheses give the numerical integration uncertainties in the last digit(s). The other uncertainties correspond to the envelope obtained by varying independently the renormalisation and factorisation scales around the central value $$1/2<\mu _{R,F}/\mu _\mathrm{0}<2$$ with $$\mu _0=m_W$$. NLO QCD corrections contribute constructively for the SM case, but destructively for the HD cases, although the global $$K$$-factors are rather mild. The uncertainties in the HD scenarios, especially for the derivative operator (HDder), are larger than that in the SM case. Manifestly, the uncertainties are significantly reduced going from LO to NLO.

**Table 4:** VBF total cross sections with scale uncertainties and corresponding $$K$$-factors at LHC 8TeV for various scenarios

Scenario	$$\sigma _\mathrm{LO}$$ (fb)	$$\sigma _\mathrm{NLO}$$ (fb)	$$K$$
$$0^+$$(SM)	1509(1) $${}^{+4.7~\%}_{-4.4~\%}$$	1633(2) $${}^{+2.0~\%}_{-1.5~\%}$$	1.08
$$0^+$$(HD)	69.66(6) $${}^{+7.5~\%}_{-6.6~\%}$$	67.08(13) $${}^{+2.2~\%}_{-2.3~\%}$$	0.96
$$0^+$$(HDder)	721.9(6) $${}^{+11.0~\%}_{-9.0~\%}$$	684.9(1.5) $${}^{+2.3~\%}_{-2.8~\%}$$	0.95
$$0^+$$(SM$$+$$HD)	3065(2) $${}^{+5.6~\%}_{-5.1~\%}$$	3144(5) $${}^{+1.6~\%}_{-1.1~\%}$$	1.03
$$0^-$$(HD)	57.10(4) $${}^{+7.7~\%}_{-6.7~\%}$$	55.24(11) $${}^{+2.1~\%}_{-2.5~\%}$$	0.97
$$0^\pm $$(HD)	63.46(5) $${}^{+7.6~\%}_{-6.7~\%}$$	61.07(13) $${}^{+2.3~\%}_{-2.0~\%}$$	0.96

For the studies on the distributions, we require the presence of at least two reconstructed jets with

5 $$\begin{aligned} p_T^j>25 \mathrm{GeV}\,,\quad |\eta ^j|<4.5\,. \end{aligned}$$

In addition, we simulate a dedicated VBF selection by imposing an invariant mass cut on the two leading jets,

6 $$\begin{aligned} m(j_1,j_2)>500\,\mathrm{GeV}\,. \end{aligned}$$

As is well known, such a cut has the scope to minimise the contributions from gluon fusion and allow one to extract VBF couplings. We note that we do not put the rapidity separation cut, although this is the common VBF cut, since $$\varDelta \eta (j_1,j_2)$$ itself is a powerful observable to determine the $$HVV$$ structure in VBF production [48, 85].

**Fig. 1:**
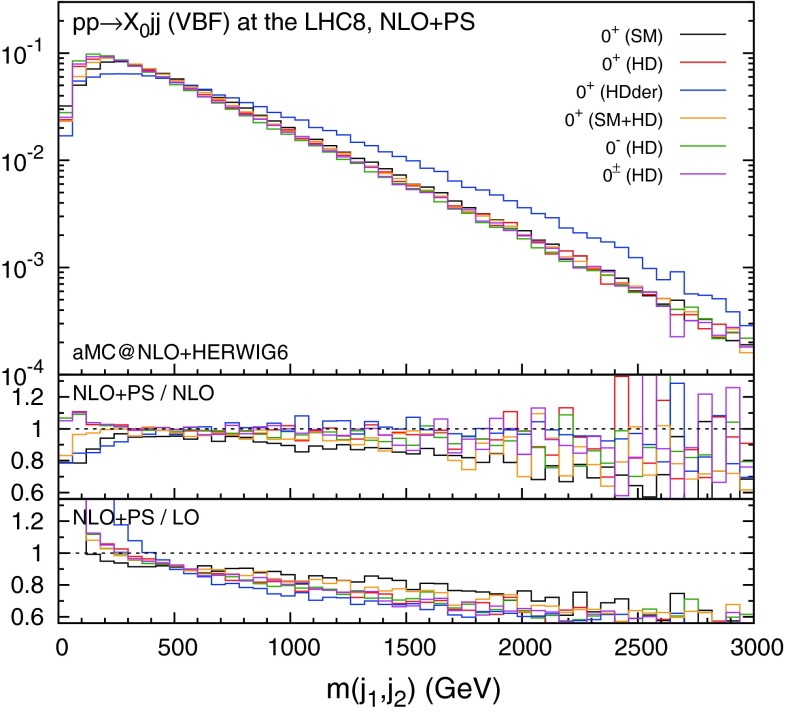
Distribution for the invariant mass of the two leading jets in VBF production with the acceptance cuts. The *histograms* in the main plot are normalised to unity

We start by showing the invariant mass distribution of the two leading jets in Fig. 1 for the six scenarios of Table 3, where the minimal detector cuts in Eq. (5) are applied. With the exception of the scenario featuring the derivative operator (HDder), the distributions are all very similar. This means that the invariant mass cut in Eq. (6), which is imposed in typical VBF selections, acts in a similar way on all scenarios.

The lowest inset in Fig. 1 is the ratio of NLO+PS to LO results, while the middle one shows the ratio of NLO+PS to pure NLO. NLO+PS corrections modify in a consistent way LO parton-level predictions with major effects at high invariant mass, *i.e.*, the QCD corrections tend to make the tagging jets softer. In addition, the parton shower affects both the lower and the higher invariant mass regions.

**Fig. 2:**
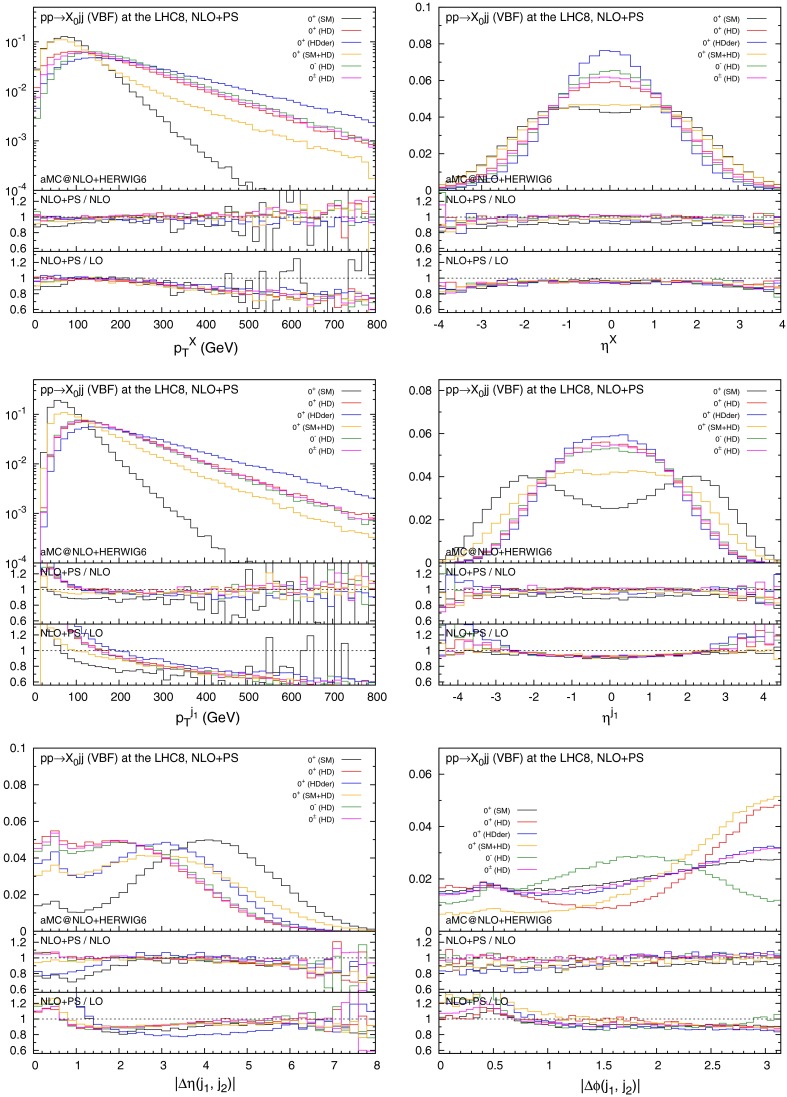
Distributions for $$p_T^X$$, $$\eta ^X$$, $$p_T^{j_1}$$, $$\eta ^{j_1}$$, $$\varDelta \eta (j_1,j_2)$$, and $$\varDelta \phi (j_1,j_2)$$ in VBF with the acceptance cuts for the jets. The *histograms* in the main plots are normalised to unity

**Fig. 3:**
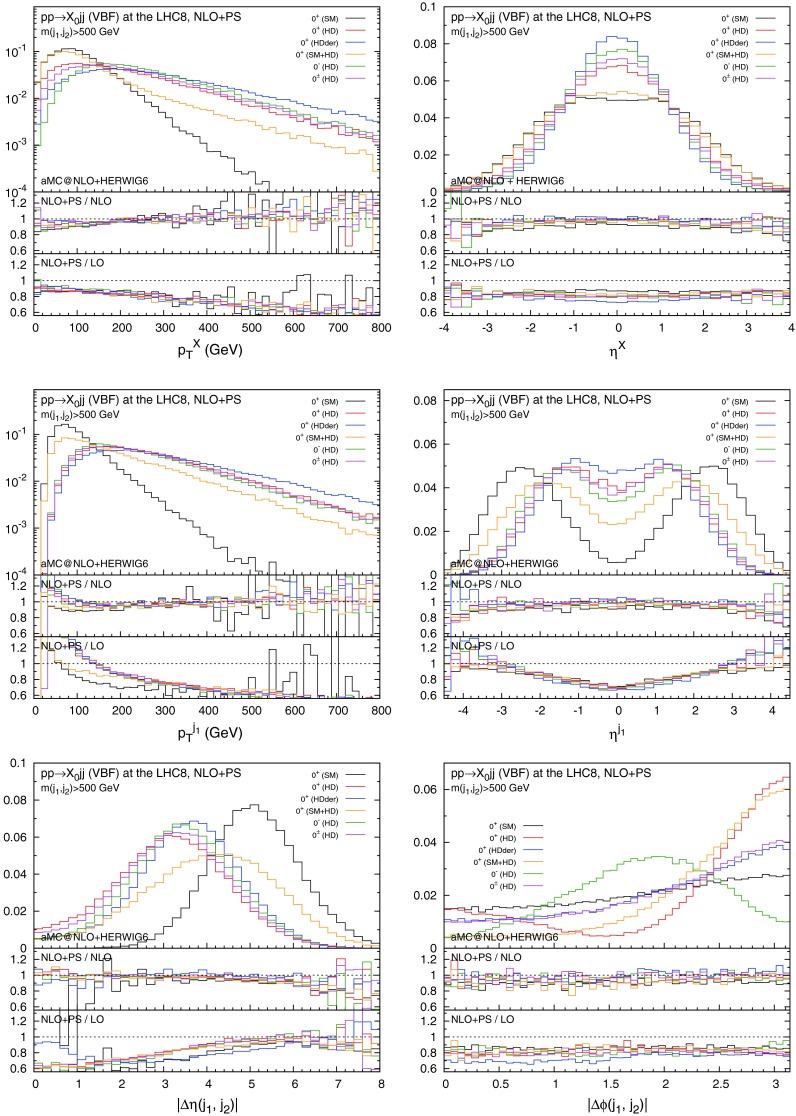
Same as Fig. 2, but with the additional VBF cut in Eq. (6)

Figures 2 and 3 collect key plots for the $$X_0$$ and the hardest jet distributions, as well as the rapidity and azimuthal separation of the two leading jets. In Fig. 2 only the acceptance cuts in Eq. (5) are imposed, while in Fig. 3 the additional VBF cut in Eq. (6) is applied. As one can see, the invariant mass cut effectively suppresses the central jet activity, especially for the SM case, while the difference of the distributions among the different scenarios becomes more pronounced.

The unitarity violating behaviour of the higher-dimensional interactions, especially for $$0^+$$(HDder), clearly manifests itself in the transverse momentum distributions for the $$X_0$$ and the jets. The rapidity distribution of the tagging jets displays the fact that in the case of higher-dimensional interactions the jets as a result are much more central than in the SM case. The same glaring difference appears in the azimuthal correlations between the jets which offer clear handle to discriminate about different interactions type and parity assignments.

In all cases NLO corrections are relevant and cannot be described by an overall $$K$$-factor. Moreover, their impact depends on the applied cuts. Apart from regions in phase space where the jets end up close and therefore are sensitive to NLO/jet reconstruction effects, the parton-shower effect on the shapes is very minor, especially after the VBF cut.

## Vector-boson associated production

Predictions for Higgs production in association with a weak vector boson in the SM are known up to NNLO accuracy [90–92], including EW corrections [93, 94]. NLO+PS results can be obtained via (a)MC@NLO [95, 96] and the POWHEG BOX [97]. Many phenomenological studies on Higgs spin, parity and couplings are available in the literature [48, 88, 98–105]. In this section we present the first predictions for EFT interactions including NLO corrections in QCD interfaced with a parton shower in the VH process.

The code and events for VH production at hadron colliders can be automatically generated by issuing the following commands:
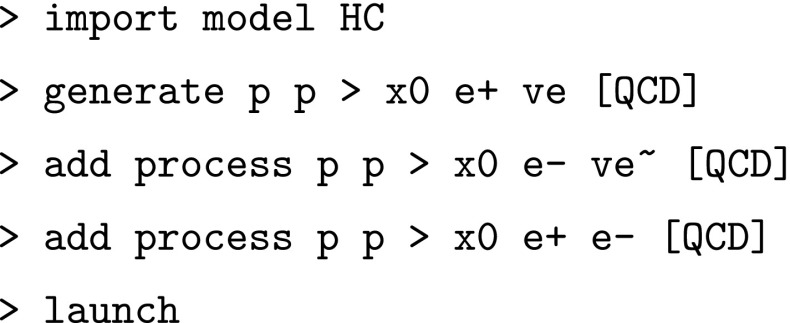
 Note that the $W,Z$ decays are performed at the level of the matrix elements and therefore all spin correlations are kept exactly. Again, as in Sect. 3, we do not consider contributions involving the $X_0\gamma \gamma$ and $X_0Z\gamma$ vertices.

Results for total cross sections (without any cuts) at LO and NLO accuracy and corresponding $K$-factors for the six scenarios defined in Table 3 are collected in Tables 5, 6 and 7 for $pp\rightarrow W^+H$, $W^-H$ and $ZH$, respectively, including the $W/Z$ decay branching ratio into a lepton pair. As in the VBF case, the uncertainties correspond to the envelope of independently varying the renormalisation and factorisation scales around the central value $1/2<\mu _{R,F}/\mu _\mathrm{0}<2$ with $\mu _0=m_\mathrm{VH}$. Apart from the case of the SM for which the uncertainties are accidentally small at LO, the results at NLO display an improved stability. Quite interestingly all $K$-factors are found to be around 1.3 for all the scenarios, with a tiny difference among the processes due to the different initial states. We note that the cancellation of the $s$-channel vector-boson propagator due to the derivative in the higher-dimensional scenarios results in the rather large cross section in spite of the $\varLambda =1$ TeV cutoff (except for the $0^+$(SM+HD) scenario, where $\varLambda =v=246$ GeV).

**Table 5:** $pp\rightarrow H(W^+\rightarrow e^+\nu _e)$ total cross sections with scale uncertainties and corresponding $K$-factors at LHC 8 TeV for various scenarios

Scenario	$\sigma _\mathrm{LO}$ (fb)	$\sigma _\mathrm{NLO}$ (fb)	$K$
$0^+$(SM)	39.58(3) ${}^{+0.1~\%}_{-0.6~\%}$	51.22(5) ${}^{+2.2~\%}_{-1.8~\%}$	1.29
$0^+$(HD)	13.51(1) ${}^{+1.5~\%}_{-1.7~\%}$	17.51(1) ${}^{+1.9~\%}_{-1.3~\%}$	1.30
$0^+$(HDder)	324.2(2) ${}^{+4.7~\%}_{-4.3~\%}$	416.1(4) ${}^{+2.3~\%}_{-2.1~\%}$	1.28
$0^+$(SM$+$HD)	118.8(1) ${}^{+3.0~\%}_{-2.9~\%}$	154.2(1) ${}^{+1.8~\%}_{-1.6~\%}$	1.30
$0^-$(HD)	8.386(7) ${}^{+2.6~\%}_{-2.6~\%}$	10.89(1) ${}^{+1.8~\%}_{-1.5~\%}$	1.30
$0^\pm$(HD)	10.96(1) ${}^{+1.9~\%}_{-2.1~\%}$	14.22(1) ${}^{+1.8~\%}_{-1.3~\%}$	1.30

**Table 6:** Same as Table 5, but for $pp\rightarrow H(W^-\rightarrow e^-\bar{\nu }_e)$

Scenario	$\sigma _\mathrm{LO}$ (fb)	$\sigma _\mathrm{NLO}$ (fb)	$K$
$0^+$(SM)	22.46(1) ${}^{+0.0~\%}_{-0.6~\%}$	29.86(3) ${}^{+2.3~\%}_{-1.8~\%}$	1.33
$0^+$(HD)	7.009(5) ${}^{+1.4~\%}_{-1.7~\%}$	9.355(9) ${}^{+1.9~\%}_{-1.3~\%}$	1.34
$0^+$(HDder)	145.7(1) ${}^{+4.1~\%}_{-3.9~\%}$	193.8(1) ${}^{+2.1~\%}_{-1.9~\%}$	1.33
$0^+$(SM$+$HD)	57.90(5) ${}^{+2.8~\%}_{-2.9~\%}$	77.31(8) ${}^{+1.8~\%}_{-1.6~\%}$	1.34
$0^-$(HD)	4.151(3) ${}^{+2.5~\%}_{-2.6~\%}$	5.550(5) ${}^{+1.7~\%}_{-1.4~\%}$	1.34
$0^\pm$(HD)	5.583(4) ${}^{+1.8~\%}_{-2.0~\%}$	7.445(7) ${}^{+1.8~\%}_{-1.3~\%}$	1.33

**Table 7:** Same as Table 5, but for $pp\rightarrow H(Z\rightarrow e^+e^-)$

Scenario	$\sigma _\mathrm{LO}$ (fb)	$\sigma _\mathrm{NLO}$ (fb)	$K$
$0^+$(SM)	10.13(1) ${}^{+0.0~\%}_{-0.5~\%}$	13.24(1) ${}^{+2.2~\%}_{-1.7~\%}$	1.31
$0^+$(HD)	2.638(2) ${}^{+1.4~\%}_{-1.7~\%}$	3.461(3) ${}^{+1.9~\%}_{-1.3~\%}$	1.31
$0^+$(HDder)	48.61(4) ${}^{+4.2~\%}_{-3.9~\%}$	63.59(5) ${}^{+2.1~\%}_{-1.9~\%}$	1.31
$0^+$(SM$+$HD)	19.95(1) ${}^{+3.1~\%}_{-3.1~\%}$	26.24(2) ${}^{+1.8~\%}_{-1.6~\%}$	1.32
$0^-$(HD)	1.480(1) ${}^{+2.6~\%}_{-2.7~\%}$	1.952(1) ${}^{+1.7~\%}_{-1.5~\%}$	1.32
$0^\pm$(HD)	2.061(1) ${}^{+1.9~\%}_{-2.0~\%}$	2.705(2) ${}^{+1.8~\%}_{-1.3~\%}$	1.31

**Fig. 4:**
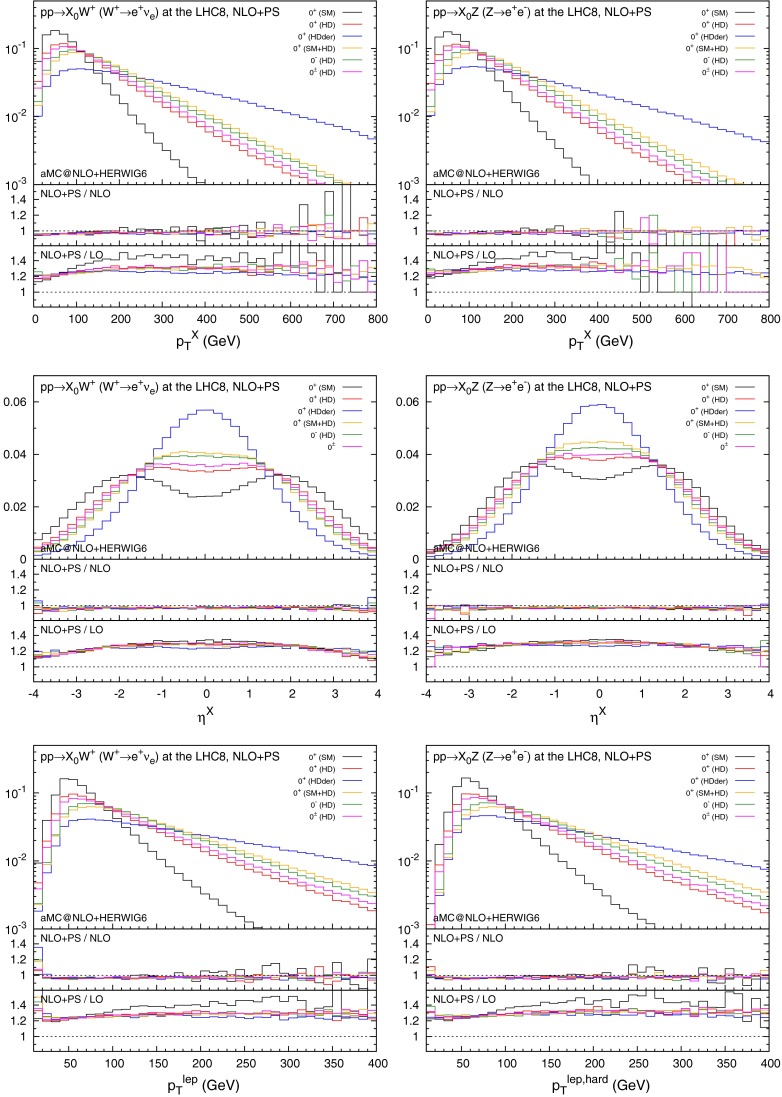
Distributions for $p_T^X$, $\eta ^X$, and $p_T^{\ell }$ in $W^+H$ (*left*) and in $ZH$ (*right*) production with the acceptance cuts for the lepton(s). The *histograms* in the main plots are normalised to unity

We then show, see Fig. 4, the distributions for the several inclusive variables with minimal cuts on the charged lepton(s):

7 $$\begin{aligned} p_T^\ell >10\,\mathrm{GeV}\,,\quad |\eta ^\ell |<2.5\,, \end{aligned}$$

for $W^+H$ and $ZH$ production (distributions for $W^-H$ are very similar to $W^+H$ and we do not display them).

The results for $W$ and $Z$ display very similar features. The scenarios that include contributions from higher-dimensional operators show harder $p_T$ spectra. This is even more pronounced in the case of the derivative operator (HDder). This fact is also reflected in the shape of the rapidity distributions, *i.e.*, the harder $p_T$ spectra correspond to a more central rapidity for the VH scattering.

As in Sect. 3, the ratios of NLO+PS to LO (NLO) results are presented in the lowest (middle) inset in Fig. 4. NLO+PS effects are quite important when compared with fixed-order LO predictions, and, in many cases, they cannot be accounted for by applying an overall $K$-factor. Conversely, NLO+PS distributions are in almost perfect agreement with fixed-order NLO predictions, witnessing small effects genuinely due to the parton shower.

**Fig. 5:**
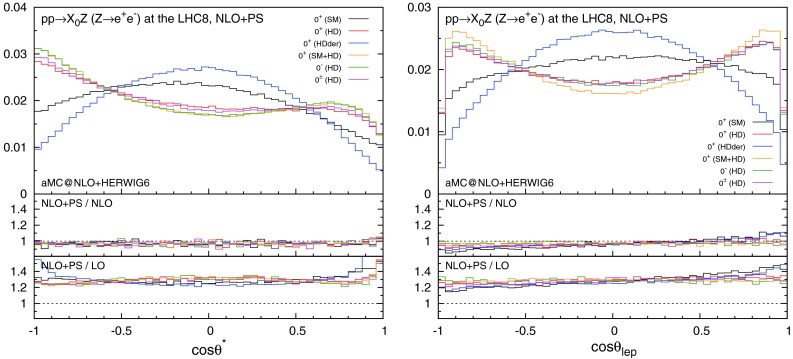
Distributions for $\cos \theta ^*$ and $\cos \theta _{\ell }$ in $ZH$ with the acceptance cuts for the leptons. The *histograms* in the main plots are normalised to unity

In Fig. 5 we show the polar angle distributions in $ZH$ production. $\cos \theta ^*$ is defined as the angle between the intermediate $Z^*$ momentum and the reconstructed $Z$ in the $Z^*$ rest frame, while $\cos \theta _{\ell }$ is the lepton angle along the $Z$ momentum in the $Z$ rest frame. In this case, NLO+PS corrections do not affect the $\cos {\theta ^*}$ distributions significantly, while those of $\cos {\theta _\ell }$ are mildly modified. We note that the asymmetry of the $\cos \theta ^*$ distribution is due to the cuts on the leptons.

## Summary

We have studied higher-order QCD effects for various spin-0 hypotheses in VBF and VH production, obtained in a fully automatic way via the model implementation in FeynRules and event generation at NLO accuracy in the MadGraph5_aMC@NLO framework. Our approach to the Higgs characterisation is based on an EFT that takes into account all relevant operators up to dimension six written in terms of fields above the EWSB scale and then expressed in terms of mass eigenstates ($W,Z,\gamma$ and $H$).

We have presented illustrative distributions obtained by interfacing NLO parton-level events to the HERWIG6 parton shower. NLO corrections improve the predictions on the total cross sections by reducing the scale dependence. In addition, our simulations show that NLO+PS effects need to be accounted for to make accurate predictions on the kinematical distributions of the final state objects, such as the Higgs and the jet distributions.

We look forward to the forthcoming LHC experimental studies employing the EFT approach and NLO accurate simulations to extract accurate information on possible new physics effects in Higgs physics.
